# Solution-processable integrated CMOS circuits based on colloidal CuInSe_2_ quantum dots

**DOI:** 10.1038/s41467-020-18932-5

**Published:** 2020-10-19

**Authors:** Hyeong Jin Yun, Jaehoon Lim, Jeongkyun Roh, Darren Chi Jin Neo, Matt Law, Victor I. Klimov

**Affiliations:** 1 Chemistry Division, Los Alamos National Laboratory, Los Alamos, NM 87545 USA; 2 Department of Energy Science and Centre for Artificial Atom, Sungkyunkwan University, Natural Sciences Campus, Seobu-ro 2066, Jangan-gu, Suwon, Gyeonggi-do 16419 Republic of Korea; 3 Department of Electrical Engineering, Pusan National University, 2 Busandaehak-ro 63beon-gil, Geumjeong-gu, Busan, 46241 Republic of Korea; 4 Department of Chemistry, University of California, Irvine, 1102 Natural Sciences II, Irvine, CA 92697 USA

**Keywords:** Electronic materials, Electrical and electronic engineering, Electronic devices

## Abstract

The emerging technology of colloidal quantum dot electronics provides an opportunity for combining the advantages of well-understood inorganic semiconductors with the chemical processability of molecular systems. So far, most research on quantum dot electronic devices has focused on materials based on Pb- and Cd chalcogenides. In addition to environmental concerns associated with the presence of toxic metals, these quantum dots are not well suited for applications in CMOS circuits due to difficulties in integrating complementary *n*- and *p*-channel transistors in a common quantum dot active layer. Here, we demonstrate that by using heavy-metal-free CuInSe_2_ quantum dots, we can address the problem of toxicity and simultaneously achieve straightforward integration of complimentary devices to prepare functional CMOS circuits. Specifically, utilizing the same spin-coated layer of CuInSe_2_ quantum dots, we realize both *p*- and *n*-channel transistors and demonstrate well-behaved integrated logic circuits with low switching voltages compatible with standard CMOS electronics.

## Abstract (web summary)

Designing efficient toxic-element-free technologies in solution-processable CMOS electronics remains a challenge. Here, the authors demonstrate integrated logic CMOS circuits based on heavy-metal-free colloidal CuInSe_2_ quantum dots with low switching voltages and with degradation-free performance on month-long time scales.

## Introduction

Chemically prepared semiconductor nanocrystals, known also as colloidal quantum dots (CQDs), have been actively investigated as an emerging materials platform for solution-processable electronics with a scope of prospective applications similar to that of more mature “plastic electronics”^1–5^. As-prepared CQDs feature a crystalline inorganic semiconductor core overcoated with a shell of organic ligand molecules. The electronic structure of the CQDs is primarily defined by characteristics of the core material, while their chemical behavior is controlled by properties of surface ligands^6–9^. As a result of these hybrid organic/inorganic structural features, CQDs combine the advantages of well-understood traditional semiconductors with the chemical flexibility and processability of molecular systems. In particular, CQDs can be fabricated and then readily processed into functional devices via low-cost, easily scalable solution-based techniques^10–20^. These features make them similar to organic semiconductors and small molecules presently exploited in flexible electronics^21–23^. At the same time, CQDs offer a number of advantageous functional distinctions derived from the inorganic nature of their quantum-confined cores such as high chemical and environment stability^16,24^, size/shape-controlled electronic characteristics derived from those of parental bulk solids^25,26^, a size-tunable bandgap^27,28^, adjustable dot-to-dot coupling^29–31^, and fairly straightforward interfacing with traditional circuits^32^.

Many initial insights into charge-transport properties of CQDs have been gleaned from studies related to their applications in solar photovoltaics (PVs)^9,15,16,29,33^ and light emitting diodes (LEDs)^34–36^. In fact, advanced understanding of (photo)conductance along with the development of effective approaches for manipulating the charge-transport characteristics of CQD solids have underlined demonstrations of PVs and LEDs whose characteristics are on a par with those of devices based on organic materials^9,33–35^.

There has been considerable research on prospective applications of CQDs in microelectronics^1–4,21,22,37–39^. This work has resulted in the development of well-performing field-effect transistors (FETs) with both *n*- and *p*-type channels (NFET and PFET, respectively)^2,4,39,40^ as well as proof-of-principle demonstrations of CQD-based integrated circuits including logic-gate devices^21,37^. However, many challenges still need to be addressed to establish CQDs as a viable materials platform for practically implementing ideas of flexible electronics. One such challenge is the demonstration of complimentary NFET-PFET pairs realized with the same CQD material, as in the case of Si-based complementary metal-oxide-semiconductor (CMOS) devices. A further problem is achieving long-term stability of these devices using, for example, encapsulation techniques that shield CQDs from the environment but at the same time do not interfere with their electronic behavior. In addition, given that most of the conducted fundamental and applied studies have utilized heavy metal-based (e.g., PbS and CdSe) CQDs, another important objective is to develop alternative “electronic-grade” CQD materials based on nontoxic compounds that can enable both *n*- and *p*-channel devices.

Here, we demonstrate that the above challenges can be successfully tackled using ternary, heavy metal-free CuInSe_2_ CQDs. We prepare these materials using a single-pot, moderate-temperature (<250 °C) colloidal synthesis that produces highly crystalline CQDs coated with long organic ligands. As organic molecules represent one of the sources of instability in solid-state CQD films, we replace them with inorganic halide-based ionic species. This improves the robustness of the self-assembled CQD films and simultaneously reduces the inter-dot spacing, thereby enhancing charge carrier mobilities. By varying the ionic species, we are able to tune the energies of the CQD electronic states versus the Fermi level and, as a result, change charge-transport behavior from degenerate *p*-type to nondegenerate *p*-type and ambipolar. Further, by combing the halide-based surface treatment with controlled, moderate-temperature annealing in the presence of indium (In), we exploit strong electron-donating ability of In to switch film conductance from *p*-type to *n*-type. We also demonstrate that the electron mobility can be tuned by adjusting the annealing temperature, which helps us reduce the mismatch between electrical characteristics of the *n*- and *p*-channel devices. We further show that the *n*- and *p*-type transport characteristics are preserved and even enhanced upon device encapsulation by a thin layer of Al_2_O_3_ prepared via atomic layer deposition (ALD). As proof of the practical utility of the developed methodologies for controlling charge transport in CuInSe_2_ CQD films, we demonstrate well-behaved, low-switching-voltage (0–5 V) CQD-based CMOS devices including an inverter (NOT gate) as well as negative AND (NAND) and negative OR (NOR) logic gates. Importantly, these devices are integrated into the same underlying CQD layer prepared by spin-coating onto a substrate with prepatterned gold and indium electrodes that define, respectively, the complimentary NFETs and PFETs. This method does not require patterning of the CQD layer and allows for “programming” the device function at the stage of the deposition of FET contacts and the connecting metal circuits. This should greatly simplify future efforts on device miniaturization and practical implementation of large-scale, highly integrated CMOS circuits.

## Results

### Charge-transport properties of CuInSe_*x*_S_2−*x*_ CQDs

So far, the majority of charge-transport studies of colloidal nanostructures have focused on Cd- and Pb-chalcogenide (e.g., CdSe and PbSe(S)) CQDs, the most synthetically advanced CQD materials. The annealed CdSe CQD films, for example, have shown high *n*-type mobilities (up to ~400 cm^2^ V^−1^ s^−1^)^2^ that have been exploited for realizing excellent switching characteristics with NFETs (ON/OFF current ratio of ~10^6^)^40^ and demonstrating unipolar integrated circuits such as NFET amplifiers, ring oscillators, and NAND and NOR logic gates^21^. The realization of *p*-doped CdSe CQDs is still a serious challenge. As in the case of bulk CdSe, achieving stable *p*-doping in this case is complicated by the very low energy of valence-band states (−6.8 eV versus vacuum).

Narrower-gap PbSe(S) CQDs have a higher absolute energy of the valence band and, as result, they can readily exhibit both *n*- and *p*-type conductance^37^. The charge-transport polarity in this case can be manipulated via straightforward postsynthetic surface treatments^38–42^. However, the extreme sensitivity of doping to surface species also presents a serious problem. For example, even small levels of unintended oxygen exposure lead to strongly degenerate *p*-type behavior even in originally *n*-doped materials^4^. On the other hand, application of an ALD treatment often switches the polarity of *p*-type PbSe(S) CQDs to *n*-type^39,42^. In addition to the above problems related to their electrical performance, CQDs of both Cd- and Pb-chalcogenides contain toxic metals that may limit their prospective commercial applications.

CuInSe_*x*_S_2−*x*_ CQDs are a heavy-metal-free alternative to CdSe and PbSe(S) CQDs^43–46^. Due to their narrow bandgap (composition tunable between 1 and 1.5 eV in the bulk form), they are excellent sunlight harvesters that have been extensively explored in the context of applications in PVs^33,44^ and luminescent solar concentrators^47^. Several recent works have also investigated charge-transport properties of CuInSe_*x*_S_2−*x*_ CQDs from the standpoint of prospective applications in electronic devices^48–50^. These studies have revealed a considerable effect of internal native defects on electrical conductance not observable in PbSe(S) and CdSe CQDs. In particular, as-prepared CuInSe_*x*_S_2−*x*_ CQDs display *p*-type transport characteristics before surface treatments, which has been ascribed to a large abundance of acceptors in the form of metal vacancies (${\mathrm{V}}^{\prime}_{\mathrm{Cu}}$ and ${\mathrm{V}}^{\prime\prime\prime}_{\mathrm{In}}$) and antisite ${\mathrm{Cu}}^{\prime\prime}_{\mathrm{In}}$ defects^49–51^ (here, lattice defects are denoted using the Kröger–Vink notation^52^). The level of *p*-doping and the hole mobility can be adjusted via traditional surface treatments (with, for example, 1,2-ethanedithiol (EDT))^49,50^. On the other hand, treatment with metal ions (Cd^2+^ and In^3+^) can be used to remove acceptors by filling metal vacancies and/or substituting for lower-valency Cu^1+^ cations^49,50^, thereby switching transport to ambipolar and to *n*-type. It was also observed that electron and hole carrier mobilities could be tuned by adjusting the Se-to-S ratio^49^. In particular, increasing the relative amount of Se in the CuInSe_*x*_S_2−*x*_ CQDs resulted in a considerable boost of both carrier mobilities, which was ascribed to the reduction in the ionization energy of donor and acceptor states with decreasing CQD bandgap^49^.

### Hole transport in CuInSe_2_ CQD films

Building upon these previous observations, here we utilize CuInSe_2_ CQDs to realize complementary *n*- and *p*-channel FETs. The use of pure-phase Se-based composition allows us to take advantage of the previously observed enhancement of carrier mobilities with increasing Se content^49^ and also gives an opportunity to employ synthetic routines that do not involve 1-dodecanethiols (DDT) commonly applied in the syntheses of pure-phase CuInS_2_ and alloyed CuInSe_*x*_S_2−*x*_ (*x* < 2) CQDs^53–55^. DDT molecules act as excellent passivating ligands, but they bind too strongly to the dot surface which makes it difficult to replace them with other species for adjusting CQD charge-transport characteristics^56^. This is especially important in the context of the present study because we exploit surface exchange of the original nonpolar ligands with strongly polar halide-based anionic species to tune the CQD doping.

**Fig. 1:**
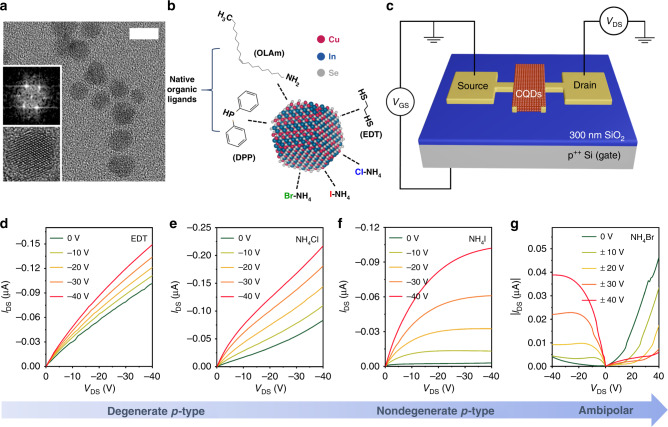
CuInSe_2_ CQD-based *p*-channel FETs. **a** A representative transmission electron microscopy (TEM) image (scale bar is 10 nm) of CuInSe_2_ colloidal quantum dots (CQDs). The CQDs have a nearly spherical shape and are characterized by an average diameter of 7.3 ± 1.9 nm. A high-resolution (HR) TEM image of an individual CQD (lower inset) and a diffractogram of a “boxed” region obtained using a fast Fourier transform (upper inset) indicate the high crystallinity of the synthesized particles. **b** A schematic depiction of a CuInSe_2_ CQD with the different types of surface ligands used in this study. As-synthesized CuInSe_2_ CQDs are capped with molecules of oleylamine (OLAm) and diphenylphosphine (DPP). For carrier transport studies, the bulky native ligands are replaced with shorter species that include ethanedithiol (EDT), NH_4_Cl, NH_4_I, and NH_4_Br. **c** A schematic diagram of a bottom-gate, bottom-contact CQD-field-effect transistor (FET). In *p*-channel FETs, source and drain electrodes are made of gold (100 nm thickness) deposited by thermal evaporation on top of a SiO_2_/*p*^++^ Si substrate (the thickness of the SiO_2_ layer is 300 nm). The channel dimensions are 3 mm (width) × 100 µm (length). CuInSe_2_ CQDs are deposited by spin-coating onto the prepatterned electrodes, and the original surface ligands are exchanged for EDT, NH_4_Cl, NH_4_I, or NH_4_Br. Output characteristics (*I*_DS_ vs. *V*_DS_) of Au-contact FETs fabricated from CuInSe_2_ CQDs with different types of surface ligands: EDT (**d**), NH_4_Cl (**e**), NH_4_I (**f**), and NH_4_Br (**g**). All devices were annealed at 180 °C for 1 h. The applied gate-source voltages (*V*_GS_) are indicated in the legends. Source data are provided as a  Source Data file.

CuInSe_2_ QDs are synthesized using a previously reported single-pot hot-injection method^10^ (see “Methods”). It results in highly crystalline, nearly spherical particles (Fig. 1a) passivated with long oleylamine (OLAm) and diphenylphosphine (DPP) ligands (Fig. 1b). The fabricated CQDs exhibit an X-ray diffraction pattern typically ascribed to the chalcopyrite crystal structure^10,57^ (Supplementary Fig. 1a). In our studies, we use CQDs with a mean diameter of 7.3 ± 1.9 nm (Fig. 1a). They show a structureless absorption spectrum (Supplementary Fig. 1b) typical of CuInSe_*x*_S_2−*x*_ CQDs. The lack of a prominent band-edge peak is due to sample polydispersity and the contribution from strong sub-band-gap absorption previously ascribed to the Cu^1+^ defects^51,53^. The elemental analysis using inductive coupled plasma-optical emission spectroscopy (ICP-OES) indicates that the Cu:In:Se atomic ratio is 0.9:1:2, suggesting that the prepared CQDs are slightly copper deficient. This has been known to promote formation of Cu vacancies that act as acceptors, leading to *p*-type conductance^58,59^.

To study charge transport in films of CuInSe_2_ CQDs, we fabricated FETs on degenerately doped *p*-type (*p*^++^) silicon wafers (Fig. 1c); see “Methods”. For transport studies, we prepared FETs with 300 nm of thermally grown silicon oxide. The 100-nm-thick metal (gold or In) source and drain electrodes were applied to the gate dielectric using thermal evaporation. The CQDs were deposited onto the substrate with the prepatterned electrodes via multiple (typically, three) spin-coating/ligand-exchange/rinsing cycles (see “Methods”); the overall thickness of the CQD film was ~100 nm. The performance of the fabricated devices was characterized in terms of a ratio of the ON and OFF drain–source currents (*I*_DS_), *β* = *I*_DS,ON_/*I*_DS,OFF_, as well as electron and hole mobilities *(μ*_e_ and *µ*_h_, respectively) derived in the linear regime from the slope of *I*_DS_ versus the gate–source voltage (*V*_GS_)^60^.

When as-synthesized CQDs are incorporated into a transistor with gold contacts (Au-FET), they show *p*-type conductance that can be modulated by the gate bias with *β* of ~4 (Supplementary Fig. 2). As discussed earlier (see also ref. ^49^), in CuInS_*x*_Se_2−*x*_ CQD solids, this *p*-type conductance arises spontaneously from a large abundance of acceptors such as cation vacancies and antisite Cu^1+^ defects.

While displaying fairly well-modulated *p*-type conductance, the films of as-synthesized CuInSe_2_ CQDs have a low hole mobility of 2.6 × 10^−5^ cm^2^ V^−1^ s^−1^, which is a result of a wide inter-dot spacing constrained by the length of the original surface ligands (Fig. 1b). To boost carrier mobilities, the original bulky ligands are usually replaced with shorter species^3^. In particular, when we treat our films with short EDT molecules (Fig. 1b), *μ*_h_ increases to a value of ~1.3 × 10^−4^ cm^2^ V^−1^ s^−1^ (Fig. 1d). However, the EDT treatment also leads to the oversupply of holes, which results in a degenerate *p*-doping behavior with a low ON/OFF current ratio (*β* = ~1.7 for *V*_DS_ = −20 V; Supplementary Fig. 3a).

Previous studies of charge transport in PbSe(S) CQD films have demonstrated that surface exchange with halide ligands (e.g., NH_4_X, where X = Cl, I, Br) reduces the degree of *p*-doping and even produces an *n*-type behavior^3,61,62^. This has been rationalized by the effect of interfacial dipoles formed by the positively charged surface metal cations and the negatively charged halide anions^63,64^. The electric field associated with these dipoles impedes extraction of an electron from the CQD, which is equivalent to lowering the energies of its electronic states versus the vacuum level. This results in an increased separation of the CQD valence-band edge from the Fermi level and in the case of strongly *p*-doped materials reduces the oversupply of holes.

Here, we exploit this effect by applying ammonium halide treatments to our CuInSe_2_ CQDs (see “Methods”). Figure 1e–g displays representative output characteristics of Au-FETs made of CQDs treated with NH_4_Cl, NH_4_I, and NH_4_Br, which leads to the replacement of the original surface passivation with anionic species Cl^–^, I^–^, and Br^–^, respectively (Fig. 1b and Supplementary Fig. 4). As in the case of EDT treatment, the surface exchange with short halide ligands increases carrier mobilities by a factor of ~10. Simultaneously, this leads to qualitative changes in the measured device characteristics. The NH_4_Cl-treated devices still show a degenerate *p*-type behavior (Fig. 1e). However, the gate-bias-induced modulation of *I*_DS_ (*β* = ~4 for *V*_DS_ = −40 V) is stronger than with the EDT-treated dots, suggesting the decreased level of  *p*-doping. The degree of doping is further reduced with the NH_4_I treatment, which leads to nearly ideal PFET characteristics (Fig. 1f) that display a good switching behavior with (*β* = ~10 for *V*_DS_ = −20 V; Supplementary Fig. 3b). The corresponding hole mobility is 1.1 × 10^−3^ cm^2^ V^−1^ s^−1^. Application of the NH_4_Br-treatment results in devices with asymmetric ambipolar characteristics suggesting an additional drop in the doping level (Fig. 1g).

To evaluate the reproducibility of the characteristics of our CQD PFETs, we have fabricated three nominally identical devices for each CQD surface treatment leading to *p*-type conductance (OLAm/DPP, EDT, NH_4_Cl, and NH_4_I). The characterization of these devices indicates good consistency of the measured characteristics. In particular, a typical device-to-device variation in the hole mobility evaluated in terms of the ratio of the standard deviation, *δμ*_h_, and the average mobility, 〈*μ*_h_〉, is from ~2% (as-synthesized OLAm/DPP-capped CQDs) to ~9% (Cl-treated CQDs); see Supplementary Table 1.

The observed trends are consistent with measurements of the effect of surface ligands on absolute energies of CQD states observed in the published literature studies^63,65^. In particular, previous measurements of PbS CQDs using ultraviolet photoelectron spectroscopy revealed that the CQD valence-band edge shifts to progressively lower energies when the ligands are switched from EDT to Cl^–^, then to I^–^, and finally to Br^–^ (ref. ^63^). In the case of a nearly constant position of the Fermi level (“pinned” by the chemical potential of the environment), this would correspond to a progressive decrease in the degree of *p*-doping, as observed in our FET measurements (Fig. 1d–g). Based on the results of the above studies, we select NH_4_I-treated dots for implementing PFET devices in the CMOS circuits discussed later in this work.

### Electron transport in CuInSe_2_ CQD films

Next, we focus on approaches for obtaining controllable levels of *n*-type doping for implementing NFETs. Previous studies of CuInSe_*x*_S_2−*x*_ CQDs demonstrate that incorporation of In leads to switching transport polarity from *p*- to *n*-type. As we discussed earlier, the *p*-type doping of as-prepared CuInSe_2_ CQDs likely originates from metal vacancies and/or antisite ${\mathrm{Cu}}^{\prime\prime}_{\mathrm{In}}$ defects. When In is incorporated into the CQD lattice by either filling a copper vacancy (i.e., creating the antisite ${\mathrm{In}}^{\cdot\cdot}_{{\mathrm{Cu}}}$ defect) or entering the interstitial space as the ${\mathrm{In}}^{\cdot\cdot\cdot}_{{\mathrm{i}}}$ defect, it acts as a compensating donor impurity^59^; and, if the amount of In is sufficiently large, the CQDs acquire *n*-type characteristics.

**Fig. 2:**
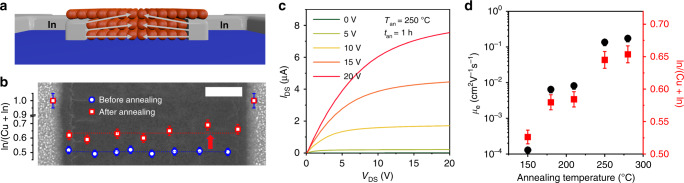
CuInSe_2_ CQD-based *n*-channel FETs. **a** *n*-channel FETs are realized by thermally annealing devices with indium source and drain contacts. During the annealing procedure indium diffuses into the CQDs wherein it acts as an *n*-dopant. **b** A top-view scanning electron microscopy (SEM) image (scale bar is 20 μm) of the In-contact CuInSe_2_ QD-FET together with the plot of the In/(Cu + In) ratio, determined by energy-dispersive X-ray spectroscopy (EDS), as a function of location along the channel before (blue circles) and after (red square) heat treatment at *T*_an_ = 250 °C for *t*_an_ = 1 h (*T*_an_ and *t*_an_ are the annealing temperature and time duration, respectively). **c** The *I*_DS_ *−* *V*_DS_ characteristics of the In-contact QD-FET made of iodide-capped CuInSe_2_ CQDs and annealed at 250 °C for 1 h; the values of applied *V*_GS_ are indicated in the legend. **d** The electron mobility (black circles) and the In/(In + Cu) ratio (red squares) of the *n*-channel In-contact FET made of CQDs treated with NH_4_I as a function of annealing temperature (annealing time *t*_an_ = 1 h). Error bars in (**b**) and (**d**) represent standard deviations determined from the measurements of three nominally identical devices. Source data are provided as a Source Data file.

Typically, In is incorporated into CQDs via thermal diffusion initiated by moderate-temperature annealing of prefabricated FETs with In source and drain electrodes^49,50^. Here, we apply this method for endowing *n*-type characteristics to our CuInSe_2_ CQDs. To implement it, we prepare FETs with In contacts (In-FETs) and then anneal them at *T*_an_ = 150–280 °C (Fig. 2a); see “Methods”. The absorption spectra of the annealed samples are nearly identical to those prior to annealing (Supplementary Fig. 1b) suggesting that the conducted heat treatment does not lead to sintering of the CQDs into a bulk-like polycrystalline film.

The effect of the annealing procedure is illustrated in Fig. 2b, which shows a top-view scanning electron microscopy (SEM) image of the device channel along with a compositional profile obtained using energy-dispersive X-ray spectroscopy (EDS) before (blue circles) and after (red squares) heat treatment at *T*_an_ = 250 °C with the annealing time (*t*_an_) of 1 h. The In content (*f*_In_) is evaluated in terms of the relative fraction of the total number of cations, *f*_In_ = In/(Cu + In). Before annealing, *f*_In_ averaged over the channel length, 〈*f*_In_〉, is 52%, and the corresponding standard deviation, *δf*_In_, is 2.5%. The obtained value of 〈*f*_In_〉 is in excellent agreement with the ICP-OES results for as-prepared CQDs according to which *f*_In_ = 1/1.9 = 0.53. Following annealing, 〈*f*_In_〉 increases to 65%. The nonuniformity in the distribution of In across the channel also increases. However, *δf*_In_ still remains within 3.2%, indicating that the annealing procedure creates a fairly uniform compositional profile throughout the entire device channel length.

In Fig. 2c, we display the *I*_DS_ − *V*_DS_ curves of the annealed In-FET (*T*_an_ = 250 °C, *t*_an_ = 1 h) made of NH_4_I-treated CQDs. The device shows excellent *n*-type characteristics with *µ*_e_ = 0.14 cm^2^ V^−1^ s^−1^, *β* of ~10^3^ (Supplementary Fig. 5a), and the electron density of ~10^17^ cm^−3^ (inferred from capacitance–voltage measurements; Supplementary Fig. 5b). The In-FETs made of EDT-treated dots also show a well-modulated *n*-type behavior with a similar *β* value of ~10^3^ (Supplementary Fig. 6). However, perhaps because of the larger length of surface ligands, the electron mobility in this case is lower by a factor of ~3 (*µ*_e_ = 0.046 cm^2^ V^−1^ s^−1^) compared to that of iodide-capped CQDs.

The implementation of CMOS circuits requires NFETs and PFETs with matching electrical characteristics, which is usually realized using *n*- and *p*-type materials with comparable carrier mobilities. Here, we exploit the strong effect of annealing temperature on *μ*_e_ of In-contact devices for reducing the mobility mismatch between *p*- and *n*-type CuInSe_2_ CQD films. Figure 2d shows that raising *T*_an_ leads to the rapid increase of the electron mobility (black circles), which correlates with the increase in the relative fraction of In in the CQDs (red squares). This observation can be explained by progressive filling (saturation) of electron traps which starts with deep intra-gap states and proceeds to shallower traps that more readily release the electrons into conducting band-edge states^49^. Based on the measurements of Fig. 2d, *T*_an_ of ~180 °C results in an electron mobility which is similar to the hole mobility of Au-FETs prepared using iodide-capped CQDs. As in the case of Au-contact PFETs, for all annealing temperatures, we observe good device-to-device reproducibility of the electron mobility realized in our In-contact NFETs (Supplementary Table 2).

### Effects of ALD treatment

The implementation of practical CMOS devices also requires a high-level of environmental stability of NFET and PFET characteristics as degradation of any element of a complementary FET pair can dramatically distort the overall behavior of the CMOS circuit. This is a serious problem in the case of unprotected CQD FETs as they often exhibit rapid degradation of their performance due to effects of the ambient environment^17,49^. In particular, the exposure of CQDs to air can lead to oxidation of their surfaces which hinders charge transport due to formation of an insulating oxide layer^5^. Furthermore, surface oxidation can alter the CQD doping, usually leading to degenerate *p*-type behavior with poor switching characteristics^3,42^.

Recently, it has been demonstrated that CQD electrical properties can be stabilized via ALD infilling of CQD films with Al_2_O_3_^39,42,49^. In addition to enhancing the stability of CQD films, this procedure also improves their charge-transport characteristics. In particular, in the case of CuInSe_*x*_S_2−*x*_ CQD FETs, the ALD treatment enhances electron and holes mobility without changing the channel polarity^49^. These beneficial outcomes of the ALD procedure have been ascribed to the passivating effect of the Al_2_O_3_ layer, which helps “heal” CQD surface defects that otherwise act as carrier traps.

**Fig. 3:**
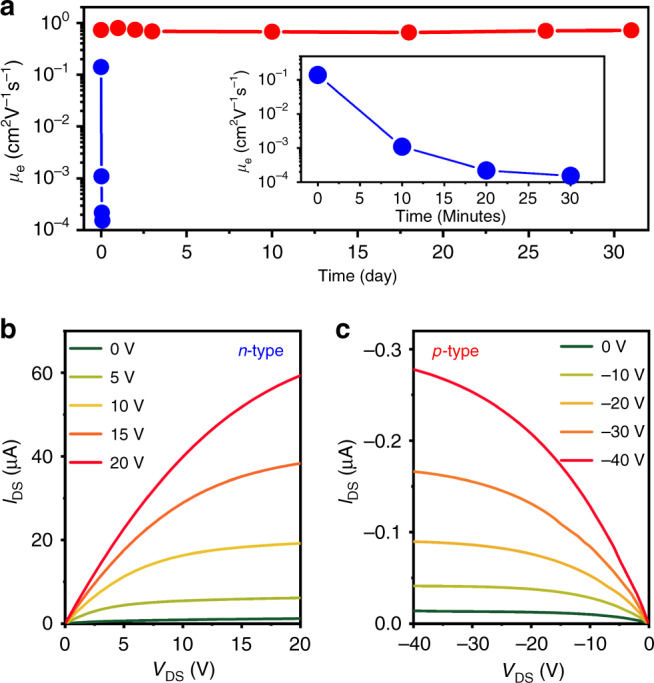
Effects of the ALD treatment on FET stability and performance. **a** Measurements of air stability of charge-transport characteristics (inferred from changes in the electron mobility) of *n*-channel devices before (blue circles) and after (red circles) encapsulation in Al_2_O_3_ using atomic layer deposition (ALD). Inset is the magnified view of the first 30 min of the degradation test for the device without ALD Al_2_O_3_. **b** The output characteristics of the *n*-channel In-contact ALD-treated FET made of iodide-capped CuInSe_2_ CQDs. **c** Same for the *p*-channel Au-contact ALD-treated FET. Source data are provided as a Source Data file.

Similar to previous reports, we also observe a considerable improvement in the performance and stability of our FETs following CQD-film infilling with Al_2_O_3_ (see “Methods”). As illustrated in Fig. 3a, without the ALD treatment, the NFET performance degrades within ~30 min in air, which manifests as a rapid drop of the electron mobility (blue symbols in the main panel and the inset of Fig. 3a). After ALD treatment, however, the electron mobility and the FET performance are completely stable for at least 30 days in air (red circles in Fig. 3a).

Importantly, the devices protected with Al_2_O_3_ also show an enhancement in both the mobility and the ON/OFF current ratio. For example, we measured *µ*_e_ = 0.70 cm^2^ V^−1^ s^−1^ and *β* = 10^4^ for the ALD-treated *n*-channel In-FET made of iodide-passivated CQDs (Fig. 3b and Supplementary Fig. 7a). Both parameters are a considerable improvement compared to devices made without ALD (*µ*_e_ = 0.14 cm^2^ V^−1^ s^−1^ and *β* = ~10^3^; Fig. 2c and Supplementary Fig. 5a). Similar improvements were also observed for the *p*-type Au-FETs (Fig. 3c and Supplementary Fig. 7b). As illustrated in Fig. 3c, in this case, the ALD infilling leads to *µ*_h_ = 3.2 × 10^−3^ cm^2^ V^−1^ s^−1^, which is ~3 times higher than the value measured before the ALD treatment. The ALD-treated PFETs also showed excellent long-term stability comparable to that of the Al_2_O_3_-encapsulated NFETs. Yet another beneficial effect of the ALD treatment is the reduction of the difference in device characteristics (hysteresis) observed for different scan directions (compare Supplementary Figs. 3b and 5a with Supplementary Fig. 7a, b). The suppression of hysteresis due to ALD was previously observed for CuInSe_2_ CQD FETs and explained by the passivating effect of the alumina coating^49^.

The ALD-infilled FETs show good reproducibility of device characteristics for both *p*- and *n*-type channels with the device-to-device variability of ~4% for *µ*_h_ and ~7% for *µ*_e_ (Supplementary Table 3). The realized mobilities are still lower than those for state-of-the-art organic FETs (*μ* > 10 cm^2^ V^−1^ s^−1^)^66,67^. However, they are comparable to those of device-grade amorphous silicon (*µ* = 0.1–1.0 cm^2^ V^−1^ s^−1^)^67,68^ and, thus, should be adequate for realizing practical devices as demonstrated in the next section.

### Fabrication and characterization of CMOS circuits

We exploit the insights gained from the conducted charge-transport studies to demonstrate integrated CMOS circuits based on CuInSe_2_ CQDs. To fabricate a specific CMOS device, we combine all required elements in a common solution-processed layer of CQDs treated with NH_4_I, which yields simultaneously good electron and hole mobilities. We start our device-related effort by demonstrating a CMOS inverter (a NOT logical gate). For proper device operation, an NFET and a PFET of a complementary transistor pair must have similar output characteristics, that is, must exhibit matching source-drain currents for the same gate voltage. To satisfy this requirement, we exploit the strong dependence of the electron mobility of In-contact NFETs on annealing temperature (Fig. 2d) for reducing disparity between *μ*_e_ and *μ*_h_. To compensate for the remaining mismatch between the NFET and PFET output characteristics, we adjust the ratio of the NFET and PFET channel widths.

**Fig. 4:**
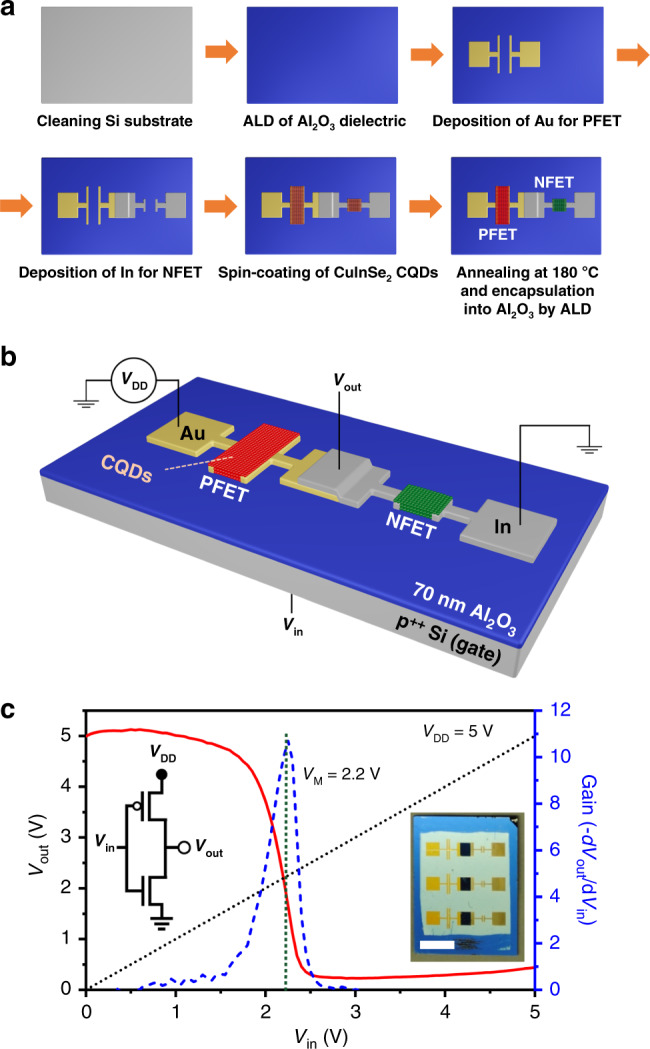
CuInSe_2_-CQD-based inverter realized using complimentary *p*- and *n*-channel FETs. **a** Processing steps used to fabricate a complementary metal-oxide-semiconductor (CMOS) inverter based on *p*- and *n*-channel CuInSe_2_ CQD FETs (PFET and NFET, respectively). After cleaning a *p*^++^ Si substrate (gate electrode), we deposited a 70-nm layer of Al_2_O_3_ by ALD to serve as a gate dielectric. Gold and then indium source and drain electrodes were deposited via thermal evaporation to define the PFET and NFET, respectively. The PFET and NFET channel widths are 3 and 1 mm, respectively. The channel lengths are the same (100 µm) for both FETs. The CuInSe_2_ CQD layer was deposited onto the substrate with the prepatterned electrodes via sequential spin-coating and ligand exchange using NH_4_I/methanol followed by washing with methanol. The device was then annealed at 180 °C to allow for indium diffusion into the CQD layer within the NFET channel. Finally, the device was encapsulated in a thin layer of Al_2_O_3_ by ALD. **b** The schematic depiction of the fabricated CQD CMOS inverter (not to scale). CQDs form a continuous film on top of a substrate with prepatterned PFET and NFET electrodes and connecting metal circuits that define the device function. The red and green areas show the region of the CQD film that act as, respectively, *p*- and *n*-type channels. **c** The voltage-transfer characteristic (VTC) of this device for *V*_DD_ = 5 V (solid red line). The dotted black line corresponds to *V*_out_ = *V*_in_. The dashed blue line is the first derivative of the VTC. Inset is a top-view photograph of the substrate with three inverters (scale bar is 5 mm). Source data are provided as a Source Data file.

The fabrication cycle used to prepare a CQD inverter is schematically depicted in Fig. 4a (see “Methods” for details). Briefly, the devices are assembled on top of a *p*^++^ Si substrate, which serves as a gate electrode. Instead of a 300 nm SiO_2_ gate-oxide layer used in the transport studies, here we utilize a 70 nm dielectric layer of Al_2_O_3_ prepared by ALD. This allows us to reduce the switching voltage to less than 5 V, that is, to values typical of standard Si CMOS circuits. To define a PFET and an NFET, we deposit pairs of, respectively, Au and In source and drain contacts by metal evaporation. Afterwards, we prepare an active CQD layer as a continuous film via a multi-step spin-coating/ligand-exchange/rinsing procedure. The fabricated devices are then annealed for 1 h at 180 °C, which enables indium diffusion into the channel defined by the In contacts and thereby produces *n*-type transport. Finally, the entire structure is encapsulated into Al_2_O_3_ using ALD. Importantly, the preparation of the inverters as well as other CMOS circuits described in this study does not require patterning of the CQD layer as the device structure and, correspondingly, its function are fully defined at the stage of the deposition of the underlying NFET and PFET electrodes and the connecting metal circuits.

The above procedures lead to NFETs whose mobility is approximately three times higher than that of PFETs. To compensate for this difference, we use an asymmetric inverter geometry wherein the ratio of the PFET and NFET channel widths is 3-to-1 (see “Methods”). This leads to good match between the NFET and PFET output characteristics (Supplementary Fig. 8a), which is key to obtaining a well-behaved inverter with the threshold voltage (*V*_M_) close to half of the supply voltage (*V*_DD_, Fig. 4b and the left inset of Fig. 4c).

Figure 4c shows a voltage-transfer characteristic  (VTC, solid red line) of the fabricated device obtained by monitoring an output voltage (*V*_out_) as a function of input bias (*V*_in_) swept from 0 to 5 V for *V*_DD_ = 5 V. The measured curve exhibits a good switching behavior with the threshold voltage *V*_M_ = 2.2 V, which is just slightly less than *V*_DD_/2 = 2.5 V. The device also shows good noise margins of 1.39 V (low) and 2.54 V (high) (Supplementary Fig. 8b) and a fairly high gain (*G*) of ~11 (dashed blue line in Fig. 4c). The latter is considerably higher compared to values demonstrated previously for an all-NFET inverter made of fused CuInSe_2_ CQDs (*G* < 2)^48^ and is comparable to gain reported for a CMOS inverter made of PbSe CQDs (*G* ≈ 14)^37^.

**Fig. 5:**
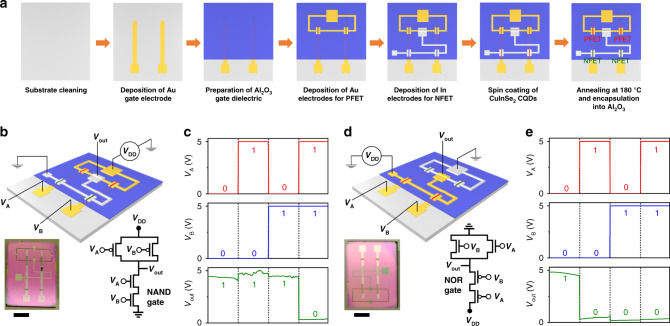
CuInSe_2_-CQD-based integrated CMOS NAND and NOR logic gates. **a** Processing steps used to fabricate a CMOS NAND gate based on CuInSe_2_ CQD PFETs and NFETs. Two separate Au gate electrodes are prepared for applying input voltages (*V*_A_ and *V*_*B*_). **b** A schematic depiction (not to scale) of the fabricated CMOS NAND gate device (top) along with its photograph (bottom left) and the circuit diagram (bottom right); *V*_DD_ = 5 V is the supply voltage and *V*_out_ is the output voltage. **c** The measured output voltage of the NAND gate for four different combinations of input signals: (*V*_A_, *V*_B_) = (0, 0), (1, 0), (0, 1), and (1, 1). **d**, **e** Same as in (**b**) and (**c**), respectively, but for the fabricated NOR logic-gate device. Scale bars in panels (**b**) and (**d**) correspond to 5 mm. Source data are provided as a Source Data file.

The above measurements indicate that the performance of the developed devices is sufficiently good for implementing more complex logic circuits. Below we provide an example of two such circuits (NAND and NOR gates) built from the CQD-based CMOS transistors. Figure 5a displays a fabrication cycle used to prepare the NAND logic gate whose diagram is depicted in Fig. 5b. In Fig. 5c, we display the results of the measurements of the device output (*V*_out_) for four different combinations of input voltages (*V*_A_ and *V*_B_). *V*_A_ and *V*_B_ are switched between 0 and 5 V; these levels correspond, respectively, to “0” (false) and “1” (true) signals. An ideal NAND gate produces a “false” signal only if both inputs are “true”; for all other input combinations, the output signal is “true”. This type of operation is indeed displayed by the fabricated device (Fig. 5c). We measure *V*_out_ = (0.3 ± 0.03) V for *V*_A_ = *V*_B_ = 5 V, and *V*_out_ = (4.5 ± 0.19) V for three other combinations of *V*_A_ and *V*_B_. Both measured output voltages are well within the noise margins of, respectively, the “false” and “true” signals (Fig. 4c) indicating that our device does perform the NAND operation in an error-free fashion. In Fig. 5d we show a schematic view of a NOR gate fabricated using complementary CQD FETs. Its measurements (Fig. 5e) indicate that it performs the expected NOR logical operation (a true output only when both inputs are false).

## Discussion

This work demonstrates that heavy metal-free CuInSe_2_ CQDs are a highly versatile materials platform for implementing *n*- and *p*-channel transistors with tunable characteristics. In particular, we demonstrate several approaches for tuning the charge-transport properties of CuInSe_2_ CQD films including the transport polarity, doping level, and carrier mobility. Through ligand exchange with halide ions, we are able to greatly enhance the hole mobility (by a factor of ~40) compared to that of films of as-prepared CQDs with original bulky organic ligands. Further, by varying the halide, we can tune the doping from highly degenerate *p*-type (Cl^−^) to nondegenerate *p*-type (I^−^) and then to ambipolar (Br^−^), which exploits the effect of surface dipoles on absolute energies of the CQD electronic states. In addition, we show that the transport polarity in halide-treated CQD films can be switched to *n*-type by the incorporation of indium implemented using moderate-temperature annealing of prefabricated FETs with In contacts. The transition from *p*- to *n*-type behavior occurs due to the compensating effect of donor states created by In ions incorporated either as interstitial (${\mathrm{In}}^{\cdot\cdot\cdot}_i$) or substitutional (${\mathrm{In}}^{\cdot\cdot}_{\mathrm{Cu}}$) defects. We also show that the electron mobility of the In-treated CQD films can be tuned over three orders of magnitude by varying the annealing temperature. We use this capability to reduce the mismatch between *μ*_e_ and *μ*_h_, and thereby simplifying practical implementation of CMOS circuits. Finally, we apply these insights to demonstrate complementary *n*- and *p*-channel transistors and CMOS logic gates (NOT, NAND, and NOR) switchable using CMOS compatible voltage levels (0–5 V). Importantly, all elements of the demonstrated CMOS devices are incorporated into a common CQD layer deposited as a continuous film onto a substrate with a prepatterned metal circuit whose structure fully defines the device function. This approach allows for straightforward device miniaturization and integration of an arbitrary number of complementary NFETs and PFETs that can be easily defined using, respectively, indium and gold contacts. Finally, after encapsulation by an Al_2_O_3_ layer prepared by ALD, the fabricated devices exhibit degradation-free performance on month-long time scales. All of these results demonstrate a considerable potential of heavy-metal-free CuInSe_2_ CQDs in solution-processable CMOS electronics.

## Methods

### Chemicals and materials

The following chemicals were purchased and used as received. Anhydrous copper (I) chloride (CuCl, 99.99%) and In (III) chloride (InCl_3_, 99.999%) were purchased from Strem Chemicals, Inc. Selenium (Se, 99.99%), oleylamine (CH_3_(CH_2_)_7_CH = CH(CH_2_)_7_CH_2_NH_2_, OLAm, 80–90%), diphenylphosphine (Ph_2_PH, DPP, 98%), anhydrous octane (CH_3_(CH_2_)_6_CH_3_, ≥99%), ammonium iodide (NH_4_I, ≥99%), anhydrous methanol (CH_3_OH, MeOH, ≥99%), 1,2-ethanedithiol (HSCH_2_CH_2_SH, EDT, ≥98%), and (3-mercaptopropyl)trimethoxysilane (HS(CH_2_)_3_Si(OCH_3_)_3_, MPTS, 95%) were obtained from Sigma-Aldrich. Isopropyl alcohol ((CH_3_)_2_CHOH, IPA, 99.5%), toluene (C_6_H_5_CH_3_, ≥99.5%), and ethanol (C_2_H_5_OH, 95%) were purchased from Fisher Scientific. Sodium selenide (Na_2_Se, 99.8%) was obtained from Alfa Aesar. Gold (99.99%) and indium (99.99%) evaporation pellets were obtained from Kurt J. Lesker Company. Highly doped *p*^++^ Si wafers with thermally grown SiO_2_ (300 nm) were purchased from Ossila Ltd.

### Synthesis of CuInSe_2_ CQDs

In a typical reaction, a solution of OLAm/DPP-Se was prepared by dissolving 2 mmol of Se powder in a mixture of 2 mmol of DPP and 5 mL of OLAm at room temperature in a nitrogen glove box. Separately, 1 mmol of CuCl and 1 mmol of InCl_3_ were dissolved in 10 mL of OLAm loaded into a 50 mL round-bottom flask and the mixture was degassed under vacuum at 110 °C for 30 min. The temperature of the reactants was raised to 180 °C and the solution of OLAm/DPP-Se was rapidly injected into the flask. To facilitate nucleation and growth of the CuInSe_2_ CQDs, the temperature was raised to 240 °C and the reaction continued for 60 min. To stop the growth, the heating element was removed and the reaction mixture was allowed to cool. The resulting CQDs were purified by several cycles (typically, three) of dissolution in toluene and precipitation with ethanol. The purified CQDs were stored in octane under nitrogen atmosphere. The described procedure produced CQDs of an approximately spherical shape with a chalcopyrite crystal structure. For parameters of the above reaction, the CQD mean diameter (*d*) was 7.3 nm and the standard deviation was 1.9 nm (=0.26*d*).

### CQD characterization

Transmission electron microscopy (TEM) images of the synthesized CQDs were recorded using a JEOL 2010 TEM equipped with a SC1000 ORIUS charge-coupled device operating at 120 kV. Optical extinction spectra were recorded using a Perkin Elmer Lambda 950 UV/Vis/NIR spectrophotometer. Elemental analysis of the CQDs was conducted using a Shimadzu ICPE-9000 inductively coupled plasma-optical emission spectrometer. The crystal structure of the CQDs was examined with a high-resolution X-ray diffractometer (Bede D1 System, Jordan Valley Semiconductors).

### Fabrication and characterization of FETs

CQD-based FETs used in charge-transport studies were fabricated on heavily *p*-doped silicon wafers with a thermally grown 300-nm layer of SiO_2_. The substrates were cleaned by successive sonication in deionized water, acetone, and IPA, and then soaked in a 5% MPTS/IPA solution for 16 h. Following the cleaning, the residual chemicals were removed by rinsing the wafer in toluene and then sonicating in IPA for 10 min. Metal (gold or indium) source and drain contacts of ~100-nm thickness were deposited on top of the oxide layer by thermal evaporation through a shadow mask; the deposition rate was 1 Å s^−1^. The typical channel width (*W*) and length (*L*) were 3 mm and 100 µm, respectively. A CuInSe_2_ CQD film was deposited on top of a prepatterned substrate via a sequence of spin-coating/ligand-exchange/rinsing steps. CuInSe_2_ QDs dissolved in octane (concentration of ∼20 mg mL^−1^) were spin-coated onto a prepatterned substrate at 1200 rpm for 30 s. For ligand exchange with EDT, a 1% solution of EDT in MeOH was spin-coated on top of the CQD layer. For CQD re-capping with halide ligands, we conducted the same procedure using a 0.1 M solution of NH_4_X (X = Cl, I, and Br) in MeOH. During the “rinsing” step, MeOH was spin-coated onto the CQD film to remove organic residues. A single spin-coating/ligand-exchange/rinsing cycle produced a CQD film of ~35 nm thickness as assessed using an atomic force microscopy (Explorer AFM, Veeco). To prepare a ca. 100 nm CQD layer used in our devices, we repeated this procedure 3 times. Using a multi-step deposition approach, we were able to achieve a virtually complete replacement of surface ligands as indicated by comparative Fourier transform infrared spectroscopy measurements of as-prepared and ligand-exchanged CQDs (Supplementary Fig. 4). Following the CQD-film preparation, Au-contact FETs were annealed at 180 °C for 1 h to remove organic residues. The In-contact FETs were annealed for 1 h using temperature varied from 150 to 280 °C. In addition to removing the remaining organic species, this procedure resulted in the incorporation of indium into CQDs, which imparted the *n*-type transport characteristics with the electron mobility dependent on the annealing temperature. The SEM and EDS studies of the fabricated devices were conducted using a JEOL JSM-IT100 InTouchScope^TM^ with an embedded JEOL EDS system. Their electrical characteristics were measured with a semiconductor device parameter analyzer B1500A, Agilent Technologies.

### Fabrication of CMOS inverters

The CMOS inverters shown in Fig. 4a were fabricated on top of a *p*^++^ Si wafer used as an input terminal. A 70-nm layer of an Al_2_O_3_ gate dielectric was prepared by ALD using a Savannah G2 deposition system, Cambridge NanoTech. Trimethylaluminum (TMA) and H_2_O were used as precursors. The substrate temperature was 200 °C and the operating pressure was ~0.1 Torr. The pulse and purge times were 15 ms and 3 s, respectively. Complementary NFET and PFET were defined by evaporating indium and gold contacts, respectively. The channel length was the same for both the NFET and the PFET (*L* = 100 µm). The channel width of the PFET (*W* = 3 mm) was greater than that of the NFET (*W* = 1 mm) by a factor of 3. Using this asymmetric configuration, we were able to compensate for the difference in electron and hole mobilities and thereby obtain matching electrical characteristics of the NFET and the PFET in a complementary device pair. Following the preparation of the electrodes, we deposited a common active layer of NH_4_I-treated CQDs as a continuous film (no patterning) by spin-coating. The fabricated CMOS inverters were annealed at 180 °C for 1 h and encapsulated into a layer of Al_2_O_3_ using ALD.

### Fabrication of CMOS logic circuits

NAND and NOR logic-gate devices (Fig. 5) were fabricated on top of a 300-nm SiO_2_/Si *p*^++^ wafer used as an underlying substrate but not a functional gate electrode. The input gate terminals used for applying *V*_A_ and *V*_B_ voltages were prepared from gold by thermal evaporation through a shadow mask. A 70-nm thick gate dielectric layer of Al_2_O_3_ was deposited by ALD using the same protocol as in the case of CMOS inverters (see previous section). The Au and In contacts defining, respectively, complementary *p*- and *n*-channel FETs were deposited by thermal evaporation through a shadow mask. A continuous 100-nm-thick film of NH_4_I-treated CQDs was prepared by spin-coating. The fabricated devices were annealed at 180 °C for 1 h and encapsulated into a layer of Al_2_O_3_ using ALD.

### Encapsulation of devices

To protect the CQD films from the effects of the environment (in particular, from exposure to ambient oxygen) and thereby stabilize their electrical properties, the fabricated devices (FETs, inverters, and logic circuits) were encapsulated within a ~20 nm thick layer of amorphous Al_2_O_3_ prepared from TMA and H_2_O precursors via ALD in a homemade cold-wall traveling-wave system inside a nitrogen-filled glove box. The substrate temperature was 75 °C, which was well below the temperature needed for indium diffusion in In-FETs. The operating pressure was ~0.1 Torr. The pulse and the purge times were 40 ms and 90-to-120 s, respectively. The preparation of a 20-nm ALD film requires 200 such ALD cycles. The ALD procedure also resulted in infilling of Al_2_O_3_ into the CQD films which improved its charge-transport characteristics as discussed in the main text.

## Supplementary information

Supplementary Information

## Data Availability

The data that support the findings of this study are available from the corresponding author upon reasonable request. Source data are provided with this paper.

## Source data

Source Data
