# Supplementary material for: Solution-processable integrated CMOS circuits based on colloidal CuInSe2 quantum dots
Source: Nat Commun. 2020 Oct 19;11:5280. doi: 10.1038/s41467-020-18932-5 (PMC7572511; doi:10.1038/s41467-020-18932-5)
Supplement: Supplementary file 1 — Supplementary Information [file 41467_2020_18932_MOESM1_ESM.pdf]

Solution processable integrated CMOS circuits based on colloidal $CuInSe_2$ quantum dots

Yun et al.

Supplementary Information

Solution-processable integrated CMOS circuits based on colloidal $CuInSe_2$ quantum dots

Hyeong Jin Yun[1], Jaehoon Lim[1,3], Jeongkyun Roh[1,4], Darren Chi Jin Neo[2], Matt Law[2], and Victor I. Klimov[1*]

[1]Chemistry Division, Los Alamos National Laboratory, Los Alamos, New Mexico 87545, United States

[2]Department of Chemistry and Department of Chemical Engineering and Materials Science, University of California, 2127 Natural Sciences II, Irvine, California 92697, United States

[3]Department of Energy Science and Centre for Artificial Atom, Sungkyunkwan University, Natural Sciences Campus, Seobu-ro 2066, Jangan-gu, Suwon 16419, Gyeonggi-do, Republic of Korea

[4]Department of Electrical Engineering, Pusan National University, 2 Busandaehak-ro 63beon-gil, Geumjeong-gu, Busan 46241, Republic of Korea

[*]Address correspondence to klimov@lanl.gov

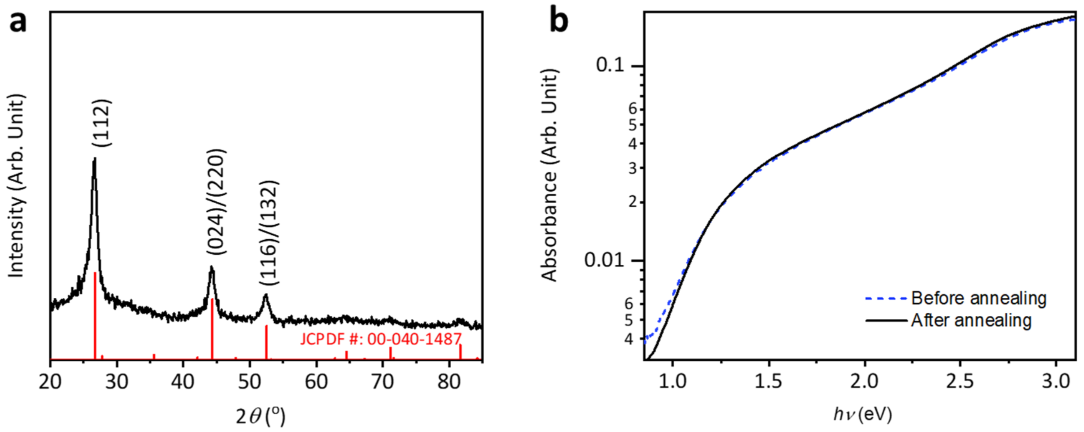

Supplementary Figure 1| XRD and spectroscopic measurements of $CuInSe_2$ CQDs. (a) The X-ray diffraction (XRD) pattern of as-synthesized $CuInSe_2$ colloidal quantum dots (CQDs) (black line). For nearly stoichiometric $CuInSe_2$, which is the case in the present study, it is usually ascribed to the chalcopyrite crystal structure[1,2] (JCPDS # 00-040-1487) (red bars). The lack of the characteristic (211) chalcopyrite peak at 2θ = 35.6° is likely due to the XRD peak broadening/smearing typical of small-size CQDs. It may also be a result of a considerable abundance of defect states such as anti-site Cu_{In}'' and $In_{Cu}^{\bullet\bullet}$ as well as copper vacancies. **(b)** Optical absorption spectra of a $CuInSe_2$ CQD film before (blue dashed line) and after (black solid line) annealing at 250 °C for 1 h. The lack of any discernable difference between the two spectra indicates that the CQDs preserve their integrity during the annealing, that is, are not sintered to form a bulk-like polycrystalline film.

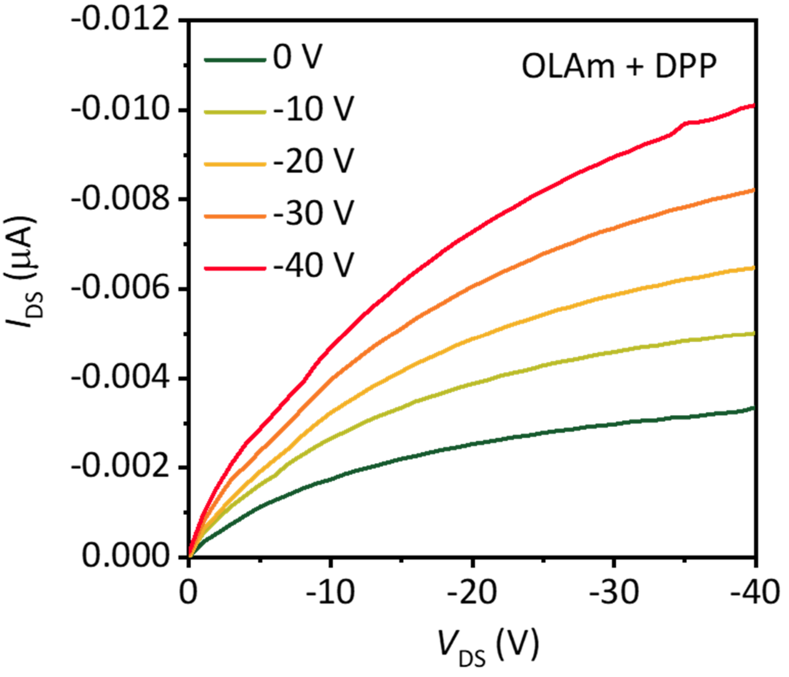

Supplementary Figure 2| Output characteristics of a *p*-type FET made of as-synthesized CQDs. The measurements of drain-to-source current (I_{DS}) versus drain-to-source voltage (V_{DS}) for a gold-contact field effect transistor (FET) fabricated from as-synthesized $CuInSe_2$ CQDs passivated with long native ligands (oleylamine (OLAm) and diphenylphosphine (DPP)); gate-to-source voltages (V_{GS}) are indicated in the legend.

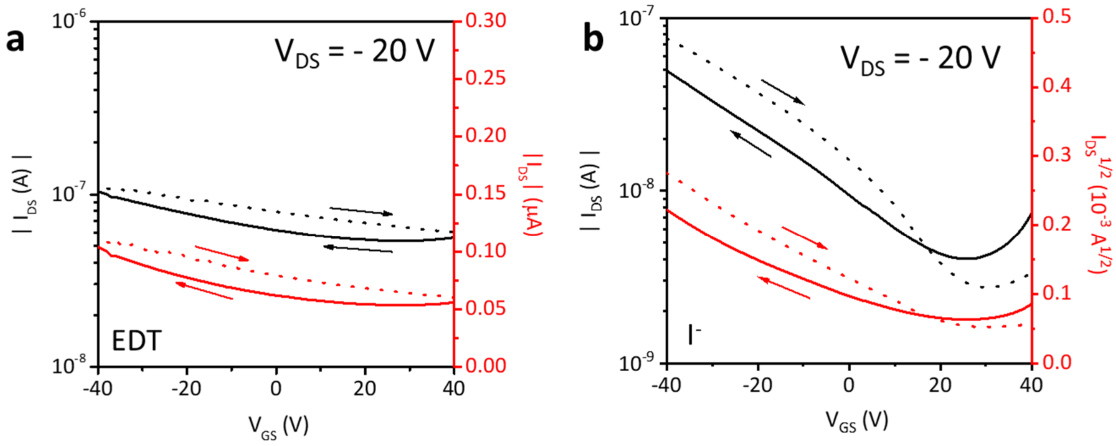

Supplementary Figure 3| Transfer characteristics of *p*-type FETs made of ligand-exchanged CQDs. The I_{DS}–V_{GS} characteristics of the *p*-type Au-contact FETs made of **(a)** EDT- and **(b)** I^--capped $CuInSe_2$ CQDs after annealing at 180 °C for 1 h. The I_{DS}–V_{GS} curves are obtained for V_{DS} = –20 V. The solid and the dotted lines are for two different scan directions.

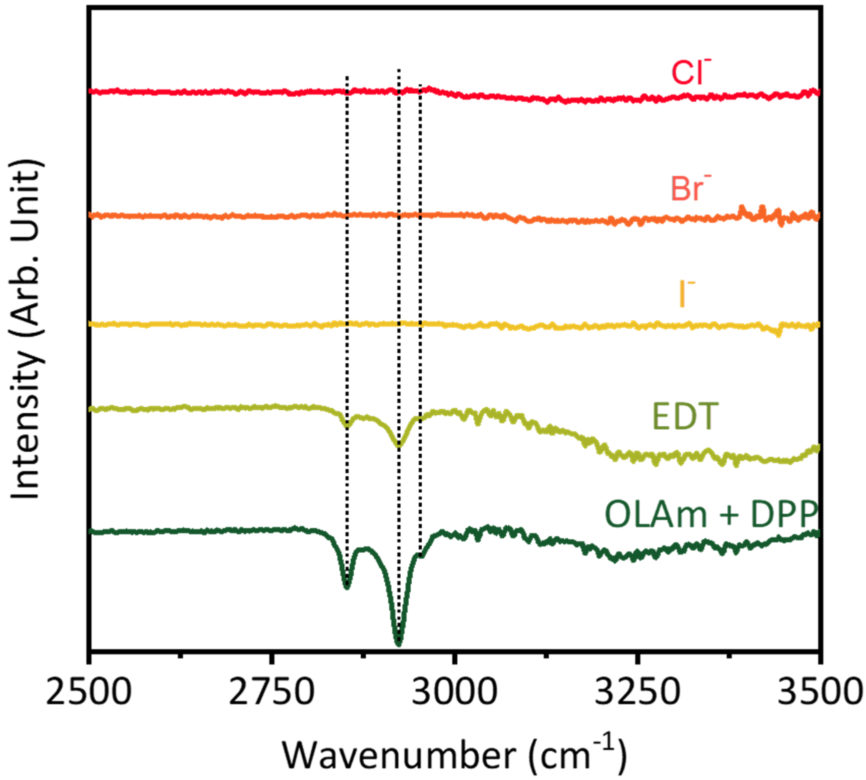

Supplementary Figure 4| Fourier transform infrared spectra of ligand-exchanged CQDs. Fourier transform infrared (FTIR) measurements reveal that the sp^3 C-H stretching peaks at 2852 (CH_2-symmetric), 2923 (CH_3-symmetric), and 2954 cm^{-1} (CH_3-asymmetric), characteristic of native OLAm and DPP ligands, are significantly reduced in amplitude after surface exchange with 1,2-ethanedithiol (EDT). These peaks become undetectable after surface exchange for anionic halide ligands indicating complete replacement of original organic surface molecules with inorganic species.

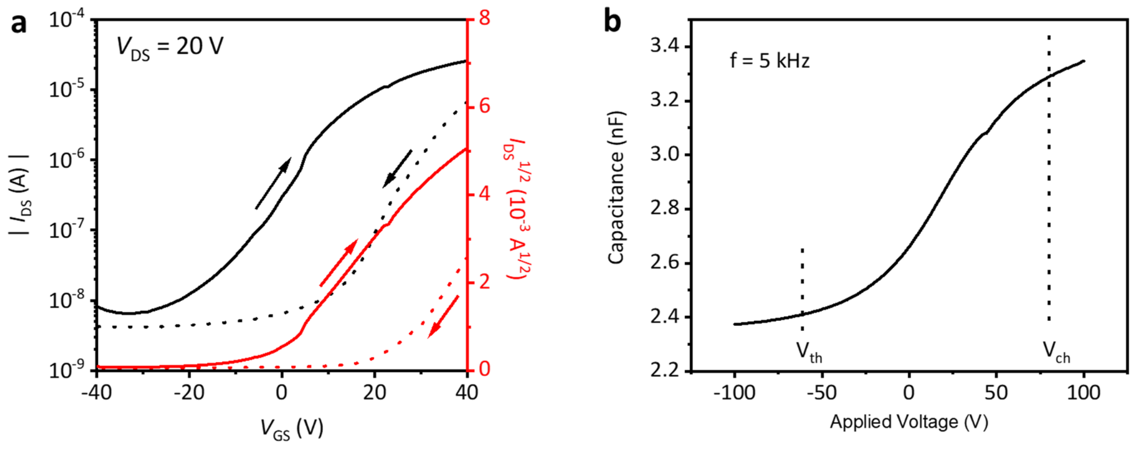

Supplementary Figure 5| Electrical characteristics of an n-type I⁻-capped-CQD FET. **(a)** The I_{DS}–V_{GS} characteristics of the In-contact CQD-FET made from I⁻-capped $CuInSe_2$ CQDs and annealed at 250 °C for 1 h. The I_{DS}–V_{GS} curves are obtained for V_{DS} = 20 V. The solid and the dotted lines are for two different scan directions. **(b)** Capacitance-voltage (C–V) characteristics of an *n*-type film of I⁻-capped $CuInSe_2$ CQDs annealed at 250 °C for 1 h. The measurement is conducted at 5 kHz on a capacitor-like device comprising a CQD layer (100 nm thickness) sandwiched between a Si substrate and a top indium contact with a 3-by-5 mm^2 area. The total injected charge is calculated from $Q = \int_{V_{th}}^{V_{ch}} C(V)dV$, where V_{th} is the threshold voltage and V_{ch} is the voltage for which the CQD film is fully charged. Based on these measurements, the doping density is ~10^{17} cm^{-3}.

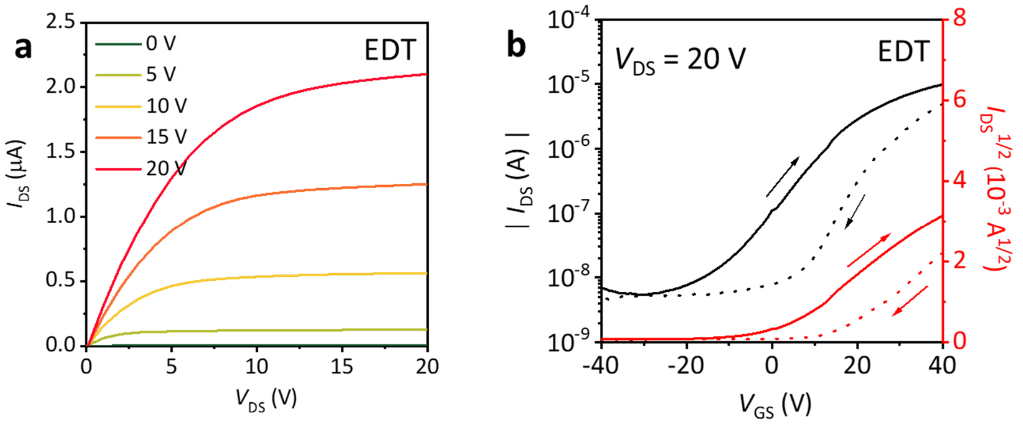

Supplementary Figure 6| Electrical characteristics of an *n*-type EDT-capped-CQD FET. (a) I_{DS}–V_{DS} and **(b)** I_{DS}–V_{GS} characteristics of the In-contact CQD-FET made of EDT-capped $CuInSe_2$ CQDs and annealed at T_{an} = 250 °C for 1 h. The I_{DS}–V_{GS} curves are obtained for V_{DS} = 20 V. The solid and the dotted lines in '**b**' are for two different scan directions.

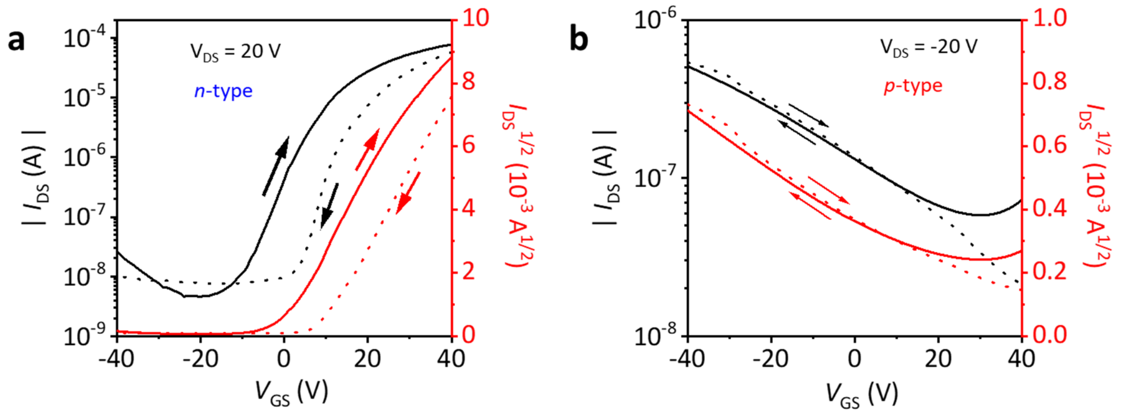

Supplementary Figure 7| Transfer characteristics of ALD-treated *n*- and *p*-type FETs (a) The I_{DS}–V_{GS} characteristics of the *n*-type In-contact FET made of I^--capped $CuInSe_2$ CQDs (T_{an} = 250 °C) and encapsulated in Al_2O_3 by atomic layer deposition (ALD). **(b)** Same for the *p*-type ALD-treated Au-contact FET.

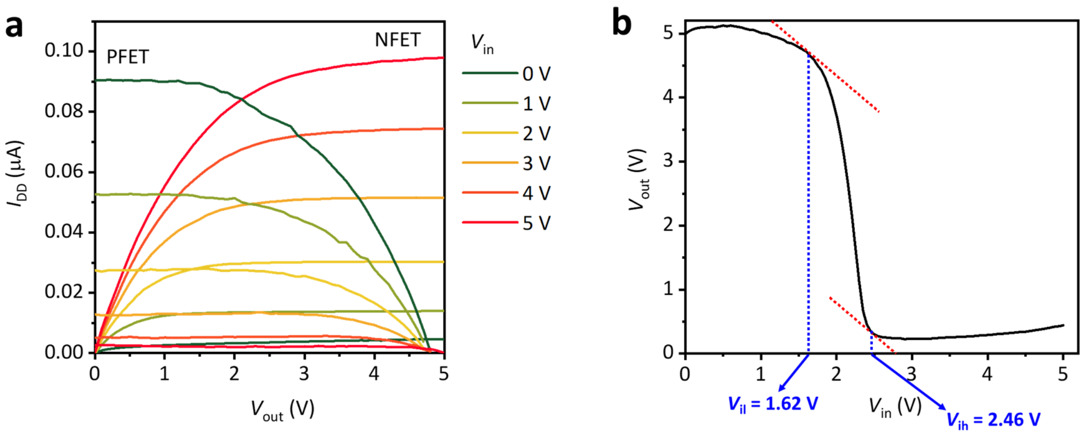

Supplementary Figure 8| Electrical characteristics of a CQD-based CMOS inverter. (a) Load curves for the PFET and NFET of the $CuInSe_2$ CQD-CMOS inverter (V_{DD} = 5 V). The input voltages (V_{in}) are indicated on the right of the plot. **(b)** The noise margin low (NML) and the noise margin high (NMH) are determined from the voltage transfer curve (VTC; black solid line) using, respectively, the expressions NML = $V_{il} - V_{ol}$ and NMH = $V_{oh} - V_{ih}$; here V_{il} (= 1.62 V) and V_{ih} (=2.46 V) are the points where the gain (dV_{out}/dV_{in}) of the VTC is equals to –1 (red dashed lines), V_{oh} (=5 V) is the maximum output voltage at which the output is "logic high", and V_{ol} (=0.23 V) is the minimum output voltage at which the output is "logic low". Based on these definitions, NML = 1.39 V and NMH = 2.54 V, which correspond to ~56% and ~100% of $V_{DD}/2$, respectively (V_{DD} = 5 V is the supply voltage).

Supplementary Table 1| Statistical analysis of hole mobilities in *p*-type FETs.

Ligand	μ_h (cm^2/Vs)	$\langle\mu_h\rangle$ (cm^2/Vs)	$\delta\mu_h$	$\delta\mu_h/\langle\mu_h\rangle$ (%)
OLAm + DPP	2.60×10^{-5}	2.59×10^{-5}	5.35×10^{-7}	2.1
	2.52×10^{-5}			
	2.65×10^{-5}			
EDT	1.30×10^{-4}	1.29×10^{-4}	6.55×10^{-6}	5.1
	1.37×10^{-4}			
	1.21×10^{-4}			
NH_4Cl	7.10×10^{-4}	7.51×10^{-4}	7.01×10^{-5}	9.3
	8.50×10^{-4}			
	6.94×10^{-4}			
NH_4I	1.10×10^{-3}	1.14×10^{-3}	7.59×10^{-5}	6.7
	1.25×10^{-3}			
	1.08×10^{-3}			

Hole mobilities (μ_h) for various Au-contact $CuInSe_2$ QD-FETs. Three nominally identical devices were fabricated and characterized for each type of ligand. Average device mobilities and standard deviations are denoted, respectively, as $\langle\mu_h\rangle$ and $\delta\mu_h$.

Supplementary Table 2| Statistical analysis of electron mobilities in *n*-type FETs.

Annealing Temperature	μ_e (cm^2/Vs)	$\langle\mu_e\rangle$ (cm^2/Vs)	$\delta\mu_e$	$\delta\mu_e/\langle\mu_e\rangle$ (%)
150 °C	1.29×10^{-4}	1.27×10^{-4}	2.09×10^{-5}	16.5
	1.52×10^{-4}			
	1.01×10^{-4}			
180 °C	6.46×10^{-3}	6.36×10^{-3}	9.87×10^{-4}	15.5
	7.51×10^{-3}			
	5.10×10^{-3}			
210 °C	8.17×10^{-3}	8.15×10^{-3}	1.06×10^{-3}	13.0
	6.92×10^{-3}			
	9.50×10^{-3}			
250 °C	1.36×10^{-1}	1.34×10^{-1}	1.52×10^{-2}	11.3
	1.52×10^{-1}			
	1.15×10^{-1}			
280 °C	1.74×10^{-1}	1.74×10^{-1}	9.39×10^{-3}	5.4
	1.62×10^{-1}			
	1.85×10^{-1}			

Electron mobilities (μ_e) for In-contact FETs based on NH_4I/MeOH-treated CQDs prepared using annealing temperatures T_{an} = 150, 180, 210, 250, and 280 °C. Three nominally identical devices were fabricated and characterized for each annealing temperature. Average device mobilities and standard deviations are denoted, respectively, as $\langle\mu_e\rangle$ and $\delta\mu_e$.

Supplementary Table 3| Statistical analysis of carrier mobilities in ALD-treated FETs.

Device Type	$\mu_{h,e}$ (cm^2/Vs)	$\langle\mu_{h,e}\rangle$ (cm^2/Vs)	$\delta\mu_{h,e}$	$\delta\mu_{h,e}/\langle\mu_{h,e}\rangle$ (%)
p-type	3.19×10^{-3}	3.18×10^{-3}	1.30×10^{-4}	4.1
	3.01×10^{-3}			
	3.33×10^{-3}			
n-type	7.03×10^{-1}	6.96×10^{-1}	5.08×10^{-2}	7.3
	7.55×10^{-1}			
	6.31×10^{-1}			

Carrier mobilities for *p*- and *n*-type ($T_{an} = 250$ °C) ALD-encapsulated FETs based on NH_4I/MeOH-treated CQDs (same notations as in Tables 1 and 2). Three nominally identical devices were fabricated for each transport polarity.

Supplementary References

1. Panthani MG, Stolle CJ, Reid DK, Rhee DJ, Harvey TB, Akhavan VA, Yu Y, Korgel BA. $CuInSe_2$ quantum dot solar cells with high open-circuit voltage. *J Phys Chem Lett* **4**, 2030-2034 (2013).

2. Yang J., et al. Copper-indium-selenide quantum dot-sensitized solar cells. *Phys. Chem. Chem. Phys.* **15**, 20517-20525 (2013).
